# Supplementary material for: Content-rich biological network constructed by mining PubMed abstracts
Source: BMC Bioinformatics. 2004 Oct 8;5:147. doi: 10.1186/1471-2105-5-147 (PMC528731; doi:10.1186/1471-2105-5-147)
Supplement: Additional File 2 — The original results of the above study (non-essential files are deleted to keep the file size under the limit set by BMC bioinformatics). [file 1471-2105-5-147-S2.bz2 › chilibotAdditionalFile2/dip10/199ID8652557E688/html/ERCC3_ERCC5.html]

 


 **ERCC3** and **ERCC5** 
  
Found 46 abstracts in PubMed, retrieved 10.  
 

 What does Google say? 
 PDF only 
| .edu only 

---

**Interactive relationship** (e.g. stimulation, inhibition, etc)

**Non-interactive relationship** (e.g. studied together, co-existance, homology, etc.)

- In multivariate logistic regression analysis, low expression of ERCC1, XPB  [ **ERCC3** ]   **ERCC3** , XPG  [ **ERCC5** ]   **ERCC5** , and CSB ERCC6 was associated with a statistically significant increased risk for SCCHN.  Ref: 11900225 Cancer, 2002
- However, cells deficient in the other NER genes, XPD, XPB  [ **ERCC3** ]  and XPG  [ **ERCC5** ] , all have the same ratio of exchanges to breaks as wild type.  Ref: 12509251 DNA Repair Amst , 2002
- RESULTS Mammalian cell lines XPB  [ **ERCC3** ] , XPD, XPF, XPG  [ **ERCC5** ] , and ERCC1 deficient in the nucleotide excision repair NER gene products show resistance to Et 743.  Ref: 11731295 Chem Biol, 2001
- CONCLUSIONS Reduced expression of ERCC1, XPB  [ **ERCC3** ]   **ERCC3** , XPG  [ **ERCC5** ]   **ERCC5** , and CSB ERCC6 is associated with a more than two fold increased risk of SCCHN.  Ref: 11900225 Cancer, 2002
- In contrast to cells bearing mutations in other components of the nucleotide excision repair apparatus XPB  [ **ERCC3** ] , XPD, XPG  [ **ERCC5** ]  and CSB, cells defective for the ERCC1 XPF structure specific nuclease are highly sensitive to cisplatin.  Ref: 12202770 Nucleic Acids Res, 2002
- Here we show that with a highly defined system of purified repair factors, DDB can greatly stimulate the excision reaction reconstituted with XPA, RPA, XPC.HR23B, TFIIH  [ **ERCC3** ] , XPF.ERCC1 and XPG  [ **ERCC5** ] , up to 17 fold for CPDs and approximately 2 fold for 6 4 photoproducts 6 4PPs, indicating that no additional factor is required for the stimulation by DDB.  Ref: 11705987 J Biol Chem, 2002
- XPA and RPA, once forming a complex at the damage site, would likely work with TFIIH  [ **ERCC3** ] , XPG  [ **ERCC5** ] , and ERCC1 XPF for dual incision.  Ref: 12486030 J Biol Chem, 2003
- RESULTS The levels of ERCC1, XPB  [ **ERCC3** ]   **ERCC3** , XPG  [ **ERCC5** ]   **ERCC5** , and CSB ERCC6 transcripts were lower in cases than in controls.  Ref: 11900225 Cancer, 2002
- Here, we provide evidence that CSB is found not only in the nucleoplasm but also in the nucleolus within a complex CSB IP 150 that contains RNA pol I, TFIIH  [ **ERCC3** ] , and XPG  [ **ERCC5** ]  and promotes efficient rRNA synthesis.  Ref: 12419226 Mol Cell, 2002
- Human nucleotide excision repair is initiated by six repair factors XPA, RPA, XPC HR23B, TFIIH  [ **ERCC3** ] , XPF ERCC1, and XPG  [ **ERCC5** ]  which sequentially assemble at sites of DNA damage and effect excision of damage containing oligonucleotides.  Ref: 12138203 Mol Cell Biol, 2002
- Core NER enzymes XPA, XPF, XPG  [ **ERCC5** ] , and TFIIH  [ **ERCC3** ]  are essential for recovery.  Ref: 12531012 DNA Repair Amst , 2002
